# Cell-Autonomous Immunity: From Cytosolic Sensing to Self-Defense

**DOI:** 10.3390/ijms26094025

**Published:** 2025-04-24

**Authors:** Danlin Han, Bozheng Zhang, Zhe Wang, Yang Mi

**Affiliations:** 1 The First Clinical Medical College, Zhengzhou University, Zhengzhou 450052, China; danlinhan0703@163.com (D.H.); bozhengzhang@163.com (B.Z.); wz18568685721@163.com (Z.W.); 2 Department of Biochemistry and Molecular Biology, School of Basic Medical Sciences, Zhengzhou University, Zhengzhou 450001, China

**Keywords:** cell-autonomous immunity, cyclic GMP-AMP synthase (cGAS)-stimulator of interferon genes (STING) pathway, the retinoic acid-inducible gene I (RIG-I)-like receptors (RLRs)-mitochondrial antiviral signaling (MAVS) axis, guanylate-binding proteins (GBPs), intracellular pathogens

## Abstract

As an evolutionarily conserved and ubiquitous mechanism of host defense, non-immune cells in vertebrates possess the intrinsic ability to autonomously detect and combat intracellular pathogens. This process, termed cell-autonomous immunity, is distinct from classical innate immunity. In this review, we comprehensively examine the defense mechanisms employed by non-immune cells in response to intracellular pathogen invasion. We provide a detailed analysis of the cytosolic sensors that recognize aberrant nucleic acids, lipopolysaccharide (LPS), and other pathogen-associated molecular patterns (PAMPs). Specifically, we elucidate the molecular mechanisms underlying key signaling pathways, including the cyclic GMP-AMP synthase (cGAS)-stimulator of interferon genes (STING) pathway, the retinoic acid-inducible gene I (RIG-I)-like receptors (RLRs)-mitochondrial antiviral signaling (MAVS) axis, and the guanylate-binding proteins (GBPs)-mediated pathway. Furthermore, we critically evaluate the involvement of these pathways in the pathogenesis of various diseases, including autoimmune disorders, inflammatory conditions, and malignancies, while highlighting their potential as therapeutic targets.

## 1. Introduction

The study of immune mechanisms has undergone a profound transformation over the centuries, marked by pivotal discoveries that have reshaped our understanding of host defense. The conceptual foundation of immunology emerged in the late 18th century with Edward Jenner’s pioneering development of the smallpox vaccine [1], which demonstrated the principle of adaptive immunity through cross-protection conferred by cowpox inoculation. Innate immunity was neglected until the late 20th century, when Charles Janeway proposed the pattern recognition theory [2], which spurred a breakthrough in innate immunity. Concurrently, Jules A. Hoffmann’s 1996 discovery that *Drosophila* Toll mutants lacked antifungal defenses [3] linked Toll to innate immunity. More recently, studies have shown that individual cells, even non-immune cells, can autonomously detect and combat intracellular pathogens [4,5]. This self-reliant defense is termed cell-autonomous immunity.

Cell-autonomous immunity may represent one of the most ancient defense mechanisms, since even single-celled organisms exhibit intrinsic defense mechanisms before the emergence of specialized immune cells. For example, bacteria resist phages through restriction-modification systems and CRISPR-Cas systems [6]. Invertebrates exhibit more complex immune mechanisms. In *Caenorhabditis elegans*, epithelial cells can autonomously induce the expression of antimicrobial peptides to combat infection. Moreover, *C. elegans* has evolved innate immune systems that employ pattern recognition receptors (PRRs) and signaling cascades, such as Toll-like receptor (TLR) and MAPK pathways, to coordinate systemic defenses [7]. Similarly, *Drosophila* has evolved defense mechanisms including autophagy and Toll innate signaling pathways [8]. However, renal tubular epithelial cells of *Drosophila* can mediate defense against bacteria by inducing nitric oxide synthase, still retaining the ability of cell-autonomous immunity [9]. For mammals, the innate immune system has evolved into a highly sophisticated and mature network of defense mechanisms, involving specialized immune cells such as macrophages and dendritic cells, as well as PRRs like TLRs and nucleotide oligomerization domain (NOD)-like receptors (NLRs), which detect pathogen-associated molecular patterns (PAMPs) [10]. In contrast, cell-autonomous immunity operates at the individual cell level, enabling each cell to independently combat pathogens through sensing invading pathogens, autophagy, and inflammasome activation. Although cell-autonomous immunity operates across most cell types, it is especially prominent in non-immune cells such as epithelial cells, endothelial cells, and fibroblasts. From unicellular organisms to vertebrates, the strategy of cellular self-defense against intracellular pathogens appears conserved and is therefore a critical layer of host defense.

From the perspectives of research history and evolution, the ancient systems of cell-autonomous immunity and innate immunity represent two distinct yet interconnected components of the host defense system. Innate immunity coordinates systemic responses through the secretion of cytokines, chemokines, and phagocytosis, and it also serves as a bridge to adaptive immunity. In contrast, cell-autonomous immunity employs intracellular pathways without depending on intercellular communication. Nonetheless, these two defense systems often collaborate, as exemplified by interferons (IFNs) produced during innate immune responses, which can induce interferon-stimulated gene (ISG) expression, thereby enhancing intracellular pathogen clearance [11]. Moreover, IFNs secreted by individual cells can also reinforce autonomous defenses through autocrine signaling. In summary, these mechanisms form a layered and complementary strategy for host defense.

**Figure 1:**
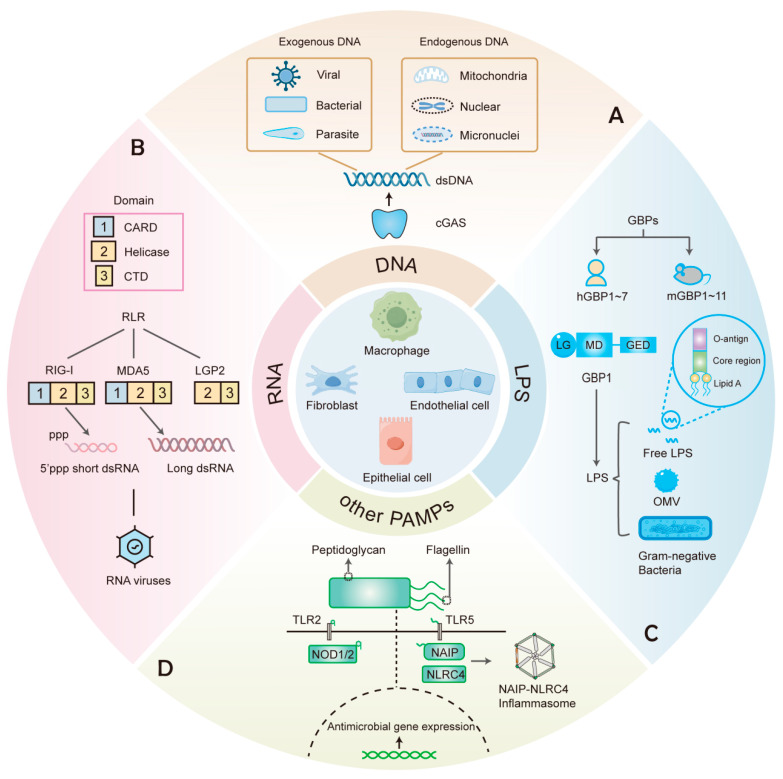
PRRs that recognize various components in cell-autonomous immunity. (**A**) cGAS recognizes dsDNA from different exogenous and endogenous sources. (**B**) RIG-I and MDA5, members of the RLRs family that contain CARD, Helicase, and CTD domains, recognize different dsRNA of viral origin. (**C**) GBPs include 7 human GBPs (hGBPs) and 11 mouse GBPs (mGBPs). GBP1 recognizes free LPS, LPS-containing OMVs, and Gram-negative bacteria. (**D**) Other PAMPs such as peptidoglycan and flagellin can elicit antimicrobial responses upon recognition by the corresponding PRRs.

Over the past two decades, research has further uncovered cytosolic sensors such as the cyclic GMP-AMP synthase (cGAS), retinoic acid-inducible gene I (RIG-I)-like receptors (RLRs), and guanylate-binding proteins (GBPs), which are recognized as key players in cell-autonomous immunity in vertebrates (Figure 1). This review aims to explore the recognition mechanisms and downstream responses against intracellular pathogens by this ancient and unique defense strategy, particularly in non-professional immune cells. Additionally, we discuss the implications of cell-autonomous immunity for various diseases.

## 2. Detection of Cytosolic DNA

The recognition of cytosolic nucleic acids is a key strategy essential for various cells to detect a wide range of pathogens. DNA was recognized as an immune stimulant long before its identification as the genetic material. Pathogen-derived DNA, such as that from DNA viruses or intracellular bacteria, as well as aberrant endogenous self-DNA, is recognized as a potential threat by host cells. Over the years, some proteins have been identified as candidates for DNA sensors (Table 1). For example, TLR9 was found to recognize endosomal DNA and stimulate immune cells to produce IFN [12]. Z-DNA-binding protein 1 (ZBP1) was first identified as a cytosolic DNA sensor but was later found to primarily recognize Z-form nucleic acids (Z-DNA/Z-RNA) and drive necroptosis [13,14]. In addition, RNA polymerase III (Pol III) can transcribe AT-rich DNA (B-form DNA) into RNA, which is then sensed by RIG-I [15]. However, both proteins are limited to specific DNA and contribute little to broad DNA sensing. Subsequent studies found that absent in melanoma 2 (AIM2) could bind DNA and lead to an inflammasome response rather than IFN induction [16]. Similarly, interferon-inducible protein 16 (IFI16) was shown not to be essential for the IFN response to human cytomegalovirus (CMV) infection by CRISPR-mediated disruption of the *IFI16* gene in primary fibroblasts [17]. Despite their contributions, these sensors exhibit limitations. Overall, these sensors are either cell type-specific, DNA sequence-specific, capable of triggering different pathways (such as AIM2 inflammasome activation), or dispensable for IFN production. The discovery of cGAS has addressed many of these limitations. cGAS is universally expressed across various cell types and provides a broadly applicable mechanism for cytosolic DNA sensing. Consequently, cGAS has emerged as a predominant sensor of cytosolic DNA recognition.

### 2.1. DNA-Sensing and the cGAS-STING Pathway

In 2012, Chen and his colleagues purified and identified the cGAS protein. They further demonstrated that cGAS was required for IFNβ induction by DNA transfection or DNA virus infection in L929 cells. Furthermore, this pioneering work validated the DNA-binding ability of cGAS through in vitro binding experiments and intracellular co-localization imaging [19]. Moreover, in an experiment where mouse lung fibroblasts were infected with herpes simplex virus (HSV) and vaccinia virus, it was found that cells from cGAS-deficient mice were unable to produce IFNβ [50]. The finding suggests that cGAS is indispensable for cytosolic DNA-induced IFNβ production. These groundbreaking discoveries reveal that cGAS is not only a novel cytosolic DNA sensor but also a central hub linking aberrant DNA detection to antiviral defense.

Structurally, cGAS belongs to the nucleotidyltransferase (NTase) family and contains a critical C-terminal NTase domain. Unlike other DNA sensors, cGAS directly binds to double-stranded DNA (dsDNA) in a sequence-independent manner [51], enabling broad-spectrum detection of DNA from various sources. cGAS can detect DNA from invading pathogens as well as mislocalized DNA within cells (Figure 1A). The entry of exogenous DNA from invading pathogens into the cytoplasm can activate cGAS. In addition to detecting DNA from DNA viruses, such as HSV and vaccinia virus, cGAS also recognizes the DNA of bacteria and parasites. For instance, research has indicated that *Mycobacterium tuberculosis* (*Mtb*) utilizes the ESX-1 secretion system to permeabilize the phagosomal membrane, enabling its DNA to access the cytosol and subsequently trigger cGAS activation [52,53]. Similarly, *Listeria monocytogenes* genomic DNA was found to induce IFN expression in a manner dependent on cGAS [54]. Moreover, it has also been found that cGAS-deficient mice exhibit higher *Plasmodium* burdens during blood-stage malaria, suggesting an important role for cGAS in controlling parasitic infections [55]. In addition to detecting exogenous DNA, cGAS is also capable of recognizing endogenous self-DNA that is abnormally localized within the cytosol [56,57]. For example, mitochondrial DNA (mtDNA) can be released into the cytoplasm by stressed mitochondria [56]. RNA viruses such as SARS-CoV-2 and dengue virus induce mtDNA leakage through mitochondrial stress, indirectly activating cGAS and resulting in antiviral defense [58,59]. Furthermore, under conditions such as DNA damage and genome instability, nuclear DNA can enter the cytoplasm, thereby activating cGAS. Micronuclei and chromatin fragments in the cytosol, important sources of immunostimulatory DNA, are often the results of abnormal DNA replication and mitotic errors [56,60].

**Figure 2:**
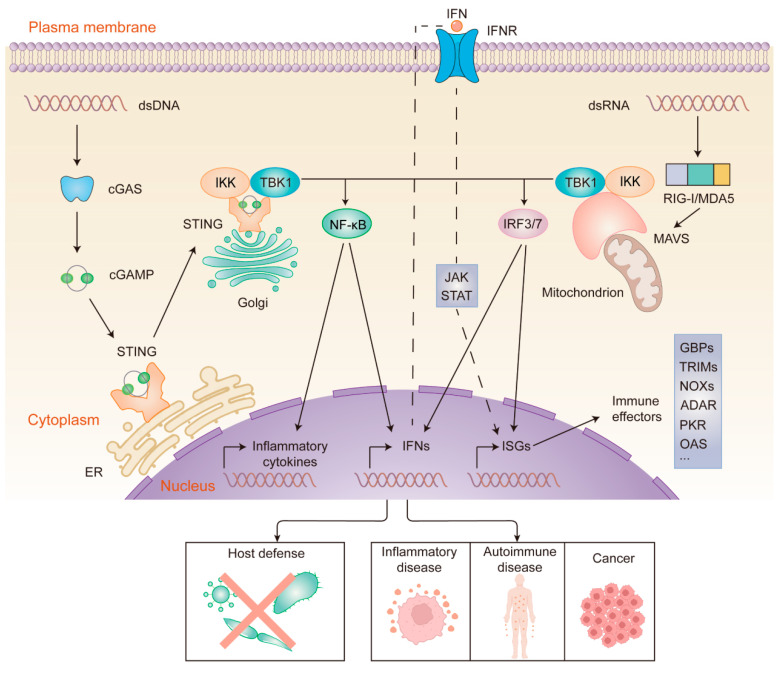
cGAS- and RIG-I/MDA5-mediated sensing and signaling of cytosolic nucleic acids. dsDNA (**left**) binds to and activates cGAS, which leads to cGAMP synthesis. Then, cGAMP binds to STING. Activated STING translocates from the ER to the Golgi. dsRNA (**right**) binds to and activates RIG-I/MDA5, which then activates MAVS at the mitochondrial membrane. Activation of STING and MAVS recruits the kinases TBK1 and IKK, resulting in the nuclear translocation of IRF3/7 and NF-κB. The phosphorylation of IRF3/7 by TBK1 dimerizes and translocates IRF3/7 to the nucleus, which induces the transcription of ISGs and genes encoding type I interferon. Likewise, IKK phosphorylation leads to the translocation of NF-κB to the nucleus, where it promotes the expression of inflammatory cytokines and IFNs. IFNs bind to IFNR in an autocrine manner, activating the JAK-STAT pathway. This ultimately leads to the expression of ISGs, which encode a variety of immune effectors. Nucleic acid sensing-driven immune responses can eliminate a wide range of pathogens, and aberrant activation of the signaling pathways has been linked to the development of certain diseases.

The cGAS-stimulator of interferon genes (STING) axis serves as a pivotal pathway that converts cytosolic DNA detection into diverse downstream antiviral and inflammatory responses. When dsDNA is detected, cGAS is activated and synthesizes a signaling molecule called 2′,3′-cyclic GMP-AMP (cGAMP). As an important endogenous second messenger, cGAMP can bind to STING, a key adaptor protein on the endoplasmic reticulum (ER) membrane, leading to its activation and translocation to the Golgi [61,62]. The process described above initiates the TANK-binding kinase 1 (TBK1)-interferon regulatory factor 3 (IRF3) signaling axis, subsequently promoting the expression of IFNs [63]. The study by Lio et al. demonstrated that CMV infection activated the cGAS-STING pathway and triggered robust type I IFN responses in primary human endothelial cells. They disrupted STING expression in endothelial cells using CRISPR-Cas9 and found that STING was critical for inducing type I IFN and limiting CMV replication [64]. STING can also activate NF-κB, which enhances the expression of pro-inflammatory cytokines and chemokines (Figure 2). In addition, STING activates autophagy by triggering the lipidation of LC3 and autophagosome formation that confers protection against pathogens. A study demonstrated that the STING (1-340) mutant, which does not induce phosphorylation of TBK1 and IRF3, suppressed HSV-1 replication in HEK293T cells. However, autophagy disruption via RavZ-mediated LC3 irreversible cleavage substantially abrogated the antiviral effects [65]. The autophagy pathway also plays an important role in resistance to bacteria such as *Mtb*, which has also been reported [66]. Another study found that STING activation resulted in lytic cell death, which activated NLRP3 through the induction of K^+^ efflux. For example, *Francisella novicida* infection could trigger the cGAS-STING-NLRP3 inflammasome axis in *CASP4* × *TRIF*-deficient human monocytes [67]. In conclusion, the multifaceted functions of the cGAS-STING pathway, including IFN induction, NF-κB activation, autophagy initiation, and inflammasome regulation, demonstrate its critical role in cell-autonomous defense.

### 2.2. Regulation of the cGAS-STING Pathway

The cGAS-STING pathway is tightly regulated to prevent either excessive activation or insufficient activity, ensuring a balanced immune response. Positive modulation of the cGAS-STING pathway is essential for mounting an effective antiviral defense. For example, TRIM56 is an IFN- and virus-inducible E3 ubiquitin ligase. A study found that TRIM56-deficient mice exhibited high susceptibility to HSV-1 infection. Mechanistically, TRIM56 catalyzes the monoubiquitination of cGAS, which promotes cGAS dimerization, enhances its DNA-binding activity, and increases its ability to synthesize cGAMP in response to HSV-1 infection [68]. Another study also showed that TRIM56 promoted the interaction between STING and TBK1 through lysine 63-linked ubiquitination of STING. This modification facilitates the recruitment and activation of TBK1, which in turn phosphorylates IRF3 to drive type I IFN production [69]. These findings highlight the promoting role of TRIM56 in the activation of antiviral responses against DNA viruses.

Conversely, the cGAS-STING pathway can be negatively regulated to prevent its overactivation. DNases localized in different subcellular compartments can prevent the overaccumulation of DNA in the cell by degrading DNA. Similarly, Beclin-1, an autophagy protein, was reported to interact with cGAS and exert a dual regulatory effect, which not only inhibited cGAMP synthesis by suppressing the cGAS NTase activity in conditions of DNA stimulation or HSV-1 infection but also enhanced cytosolic DNA degradation by autophagy [70]. These findings elucidate a crosstalk mechanism that integrates DNA-sensing and autophagy pathways. The above mechanisms ensure a balanced immune response and provide valuable insights into potential therapeutic targets for autoinflammatory and infectious diseases.

## 3. The Cytosolic RNA Sensors

Over the last two decades, candidate RNA sensors including RIG-I (also called DDX58), melanoma differentiation-associated protein 5 (MDA5), protein kinase R (PKR), 2′-5′ oligoadenylate synthetase 1 (OAS1), NLRP1/6, and ZBP1 have been reported (Table 1). Among them, RIG-I and MDA5 are members of RLRs, which exert important antiviral effects as typical cytosolic RNA sensors. RLRs stand out as the key players in cytosolic RNA sensing due to their unique evolutionary and functional advantages.

### 3.1. RLRs

In the 1970s, researchers discovered that double-stranded RNA (dsRNA) could mimic viral infection and induce IFN production, suggesting the existence of a cellular mechanism for recognizing exogenous RNA [71]. In 2001, the Alexandroff laboratory discovered that TLR3 recognized the synthetic dsRNA analog poly(I:C) and activated the NF-κB signaling pathway [72]. Subsequently, TLR7 and TLR8 were also found to recognize single-stranded RNA by using TLR-deficient mice [73]. Three members of the TLR family (TLR3/7/8) are located in nuclear endosomes and are involved in monitoring RNA viruses. They are expressed primarily in immune cells and therefore cannot account for the antiviral responses in non-immune cells. Additionally, DExD/H-box RNA helicases are ubiquitous in prokaryotes and eukaryotes, which are critical for virtually every aspect of RNA metabolism [74]. RLRs belong to the DExD/H-box RNA helicase family and play an essential role in RNA sensing. Other members of the DExD/H-box RNA helicase family, such as DDX3, DDX60, DHX9, and DHX36, may directly detect non-self RNA or may serve as co-factors in signaling pathways induced by RLRs or TLRs [75]. However, the specific mechanism of these helicases and whether they can independently recognize non-self RNA remain obscure. In 2004, Takashi Fujita’s team first reported that RIG-I mediated RNA-induced antiviral responses. Through poly(I:C)-agarose pulldown and competition assays, they found that RIG-I in 293T cells could specifically bind to dsRNA in the cytoplasm, directly triggering downstream antiviral signaling [32]. RIG-I functions as an RNA sensor in both non-immune cells (e.g., epithelial cells) and immune cells, recognizing cytosolic dsRNA and initiating antiviral responses. RIG-I is a member of the RNA helicase family and is evolutionarily conserved across various organisms, including both invertebrates and vertebrates [76]. Furthermore, a subsequent in vitro study found that MDA5 functioned similarly to RIG-I and could also recognize RNA [77]. Studies have found that RIG-I mainly recognizes 5′-triphosphate (ppp) short dsRNA, whereas MDA5 recognizes long dsRNA [34,36] (Figure 1B). Thus, RLRs can discriminate viral RNA from host RNA through unique length-dependent dsRNA, which is a mechanism absent in other RNA sensors. Some experiments have provided evidence that RIG-I and MDA5 are differentially activated by various RNA viruses. Specifically, RIG-I recognizes RNA viruses such as the Newcastle disease virus, Sendai virus, influenza virus, vesicular stomatitis virus, and Japanese encephalitis virus in various cell types such as fibroblasts. A subsequent study revealed that MDA5 is essential for detecting encephalomyocarditis virus and other picornaviruses, as MDA5-deficient cells failed to induce IFNβ upon infection, and MDA5-deficient mice exhibited severe viral susceptibility and cardiac dysfunction [33,36]. In addition to RIG-I and MDA5, laboratory of genetics and physiology 2 (LGP2) also belongs to RLRs. RIG-I and MDA5 share the common tandem caspase recruitment domain (CARD) in their N-terminal regions. However, LGP2 lacks the CARD and is thought to be a regulator of RIG-I and MDA5 [78]. In 2005, Seth et al. identified proteins containing CARD-like domains, termed mitochondrial antiviral signaling (MAVS), through a BLAST search and sequence analysis [79]. RIG-I and MDA5 interact with MAVS through CARD, thereby initiating downstream signaling in response to RNA virus infection [38].

The cytosolic cGAS-STING and RIG-I/MDA5-MAVS pathways are the most prominent pathways in nucleic acid recognition. The downstream signaling components of these two pathways partially overlap. Both STING and MAVS interact with inhibitor of NF-κB kinase (IKK)/TBK1, which causes phosphorylation of IκBα and IRF3/7, respectively, resulting in the nuclear translocation of NF-κB and IRF3/7. Eventually, two pathways are involved in inducing the expression of inflammatory cytokines and ISGs, exerting antimicrobial effects [80] (Figure 2). Importantly, some pathogens can activate both pathways. For example, a recent study found that dsRNA with a 5’ppp modification activated the RIG-I/MDA5-MAVS pathway and produced type I IFN during dengue virus infection [81]. As mentioned above, the dengue virus-induced mtDNA is recognized by cGAS. Similarly, in addition to its RNA being sensed by RIG-I, the influenza virus also induces mtDNA release in a MAVS-dependent manner, thereby activating the cGAS-STING signaling pathway [82]. Therefore, while cGAS and RLRs exhibit a high degree of specificity in pathogen recognition, the integration of their downstream signaling pathways may suggest a synergistic defense strategy against pathogens. A deeper understanding of these mechanisms will provide novel perspectives for the future treatment of viral infections, bacterial invasions, and related immunopathologic states.

In addition, non-immune and immune cells exhibit distinct strategies for nucleic acid recognition and immune responses, which together contribute to an effective host defense. Non-immune cells typically lack abundant expression of TLR and rely predominantly on cytosolic sensors to detect nucleic acid. In tissues such as the skin and gastrointestinal mucosa, where non-immune cells are highly abundant, the cell-autonomous mechanism serves as the first line of defense against extracellular pathogens. Here, pathogen clearance is achieved primarily through intracellular processes, including the induction of ISGs, autophagy, and programmed cell death, while the production of inflammatory cytokines is minimal. In contrast, immune cells express both cytosolic nucleic acid sensors and TLR3/7/8/9, enabling them to detect both extracellular and intracellular nucleic acids [33,83]. Upon pathogen detection, immune cells not only enhance their own intracellular antimicrobial responses but also secrete substantial amounts of inflammatory cytokines. These cytokines recruit additional immune cells and facilitate migration to infection sites, thereby coordinating robust innate and adaptive immune responses. In conclusion, by integrating these complementary mechanisms, the host can benefit from a multi-layered defense system. Non-immune cells provide rapid local containment of pathogens, and immune cells mobilize a systemic response to ensure thorough eradication of the invading pathogens.

### 3.2. Other RNA Sensors

Recent advances in the study of other RNA sensors have unveiled novel recognition and regulatory mechanisms, which expand our understanding of their diverse roles in cell-autonomous immunity. It was recently discovered that two NLRs, NLRP1 and NLRP6, could directly bind dsRNA and initiate the assembly of inflammasome and pyroptosis [46,48]. In addition, the OAS family of proteins is a group of template-independent RNA polymerases. These antiviral proteins detect viral infection through the binding of viral-derived dsRNA [84]. Recently, Harioudh et al. showed that OAS1, induced by IFNγ, could protect against intracellular bacterial pathogens, including *L. monocytogenes* and *F. novicida*. OAS1 was found to bind to IRF1 mRNA and facilitate its localization to ER-Golgi endomembranes, increasing the translation of IRF1 mRNA, which ultimately promoted the expression of GBPs as antimicrobial agents [39]. This finding reveals a non-classical antimicrobial role for OAS1 and extends the pathogen recognition range of this protein, which suggests the OAS family may have broader functions in cell-autonomous immunity that warrant further exploration. In parallel, GSDMB, recognized as a novel RNA sensor in the latest research, exhibited properties similar to conventional RNA sensors. In airway epithelial cells infected with respiratory viruses, GSDMB promoted the expression of ISGs and inflammation through the MAVS-TBK1 signaling pathway [49]. Future studies could further explore the therapeutic value of GSDMB in infectious or inflammatory diseases. Additionally, ZBP1 not only serves as a DNA sensor but also detects Z-RNA with Zα domains as an RNA sensor. The activation of ZBP1 triggers inflammatory responses and cell death, which are pivotal for antiviral defense [85,86]. The RNA-editing enzyme adenosine deaminase acting on RNA 1 (ADAR1) plays a crucial regulatory role by limiting the accumulation of endogenous immunostimulatory dsRNA. As early as 2012, researchers discovered the mutations in *ADAR1* led to unedited dsRNA accumulation, which was erroneously recognized by MDA5, eventually triggering the excessive production of type I IFN [87]. Recently, Zhang et al. eliminated the gene encoding ADAR1 in mouse embryo fibroblasts using a CRISPR approach and found an accumulation of endogenous Z-RNA in ADAR1-deficient cells, which subsequently activated ZBP1 [88]. Thus, ADAR1 could also function as a negative regulator for ZBP1 and is important for preventing excessive inflammation.

## 4. GBPs: Cytosolic Sensors for LPS

### 4.1. Overview of IFN-Induced GBPs

GBPs, a class of ISGs, function as both sensors and antimicrobial effectors [89]. As members of the dynamin-like GTPase family, GBPs play a critical role in fighting intracellular pathogens by sensing their presence, activating inflammasomes, and inducing cell death [90,91,92]. Typically, GBPs partition between the endomembranes and cytosol, patrolling for pathogens residing in vacuoles and microbes escaping into the cytosol. GBPs are broadly distributed among eukaryotes, spanning from plants to humans [93]. The 11 mouse GBPs (mGBPs) are encoded on chromosomes 3 and 5, while the genes encoding 7 human GBPs (hGBPs) are located on chromosome 1. Although earlier research predominantly focused on mGBPs, increasing attention has been directed toward hGBPs, which will be the primary focus of our discussion.

### 4.2. LPS Recognition and Assembly of GBP1 Defense Complex

As is known, extracellular lipopolysaccharide (LPS) can be recognized by TLR4 expressed on the cell surface. LPS, the principal component of the Gram-negative cell wall, has three structural regions: O-antigen, core polysaccharide, and lipid A (Figure 1C). Early studies demonstrated that caspase-11 (mouse) and caspase-4/5 (human) could detect intracellular LPS [94,95]. A pivotal study in 2014 showed that in Gbp^chr3^-deficient mouse macrophages, cytosolic LPS-induced pyroptosis of caspase-11 derived from *Salmonella enterica* serovar Typhimurium (*Stm*) and *Legionella pneumophila* was diminished [96], suggesting that mGBPs may participate in sensing LPS. Subsequent work in 2020 revealed that in hGBP1-deficient HeLa cells, *Stm* and transfected LPS failed to activate caspase-4 [91], establishing hGBP1 as the key initiator of LPS recognition and inflammasome activation. Notably, in this study, the data of the surface plasmon resonance test showed that purified recombinant His-GBP1 could directly bind to LPS. The above evidence suggests that GBP1 is a novel sensor that detects intracellular LPS. Currently, it has been documented that GBPs can be recruited to transfected LPS, LPS-containing bacterial outer membrane vesicles (OMVs), and invading Gram-negative bacteria [91,97] (Figure 1C).

Like other dynamin-like GTPases, the GBPs can assemble into large multiprotein complexes. Structurally, they contain an N-terminal large globular GTPase (LG) domain, a central α-helical middle domain (MD), and a C-terminal α-helical GTPase effector domain (GED) [98] (Figure 1C). GBPs have high GTPase activity to catalyze the hydrolysis of GTP and GDP, which is the energetic basis for complex formation [99]. In addition, the C-terminal CaaX motif of some GBPs is used for post-translational isoprenylation, including farnesylated (hGBP1) or geranylgeranylated (hGBP2/5). At the same time, the polybasic motif probably undergoes electrostatic interactions with negatively charged phosphates in the inner core and lipid A. The above two structures synergize to promote more GBPs to aggregate and anchor on the bacterial surface [91,100].

**Figure 3:**
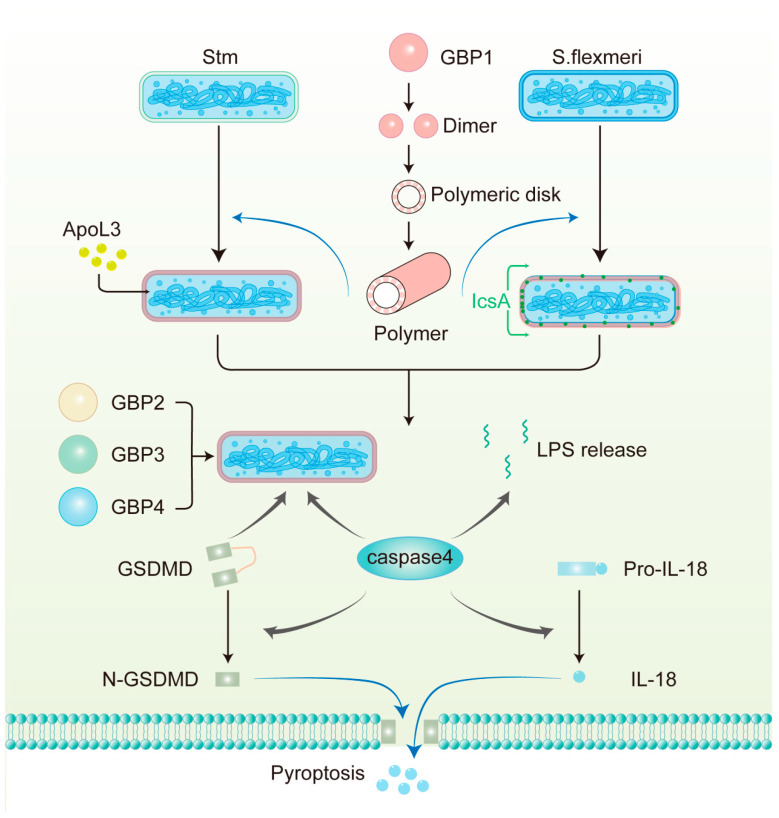
The mechanism of antimicrobial action of GBPs against Gram-negative bacteria in human epithelial cells. GBP1 polymerizes into polymers and then builds a protein coat on the surface of pathogens, including *S. flexneri*, which lacks OspC3 and IpaH9.8 and *Stm*. GBP1 disrupts the LPS barrier and allows APOL3 to reach the inner membrane to kill *Stm* directly. GBP1 also facilitates the circumferential localization of IcsA on *S. flexneri*, thereby limiting actin tail formation. GBP2-4, together with caspase-4 and GSDMD, are then recruited to the bacterial surface. GBP1-mediated LPS release from intracellular bacteria can also activate caspase-4 in the cytoplasm. Subsequently, caspase-4 cleaves full-length GSDMD to N-GSDMD, which is the active form, forming pores in the plasma membrane. Additionally, caspase-4 processes pro-IL-18 into bioactive IL-18, which is subsequently released from the cell through these pores.

In human epithelial cells, hGBP1 binds directly to the surface of Gram-negative bacteria such as *Stm* and *Shigella flexneri* [90,91] (Figure 3). An in vitro assay demonstrated that GBP1 oligomerized into polymers, which bound to the LPS on the surface of *S. flexneri* and then transitioned into a coat that encapsulated the bacterium [101]. The model provides a mechanistic explanation of how GBP1 binds to *S. flexneri*. However, whether this process occurs similarly in vivo remains to be confirmed, and it is necessary to verify the model’s applicability to other bacteria. This study also found that the GBP1 coating disrupted the O-antigen barrier, exposing lipid A to caspase-4. Another study showed that in HeLa cells, GBP1 on the surface of *Stm* or *S. flexneri* provided a platform for recruiting GBP2-4 and caspase-4. Importantly, these four GBPs acted synergistically in recruiting and activating caspase-4 [90].

Recently, MacMicking and his colleagues identified a defense complex on *Stm* inside human epithelial cells. The defense complex includes not only the previously identified GBP1-4 and caspase-4 but also full-length gasdermin D (GSDMD), which was observed to be recruited to bacteria in their study. Moreover, using cryo-electron tomography, they discovered that thousands of GBP1 dimers were vertically anchored to the outer membrane (OM) of bacteria by their extended GED domain, triggering coassembled caspase-4 activation by exposed LPS [102]. These experiments elucidate the structure of the GBP1 defense complex and the dynamic interactions among its components and provide a new dynamic perspective on GBPs in the fight against intracellular Gram-negative bacteria. Another study on *S. flexneri* showed that GBP1 promoted the release of bacterial LPS into the cytoplasm, where it activated caspase-4 [103] (Figure 3). This finding suggests that caspase-4 is not only recruited to the bacterial surface but can also be activated by free LPS in the cytoplasm, which opens a new avenue for future research.

### 4.3. GBPs Recognize Pathogens Residing Within Vacuoles

GBPs can also recognize and translocate to pathogen-containing vacuoles (PCVs), a process that depends on host components such as sugar-binding galectins and ubiquitin. For example, intact PCVs of *Toxoplasma gondii* were modified by ubiquitination. Ubiquitin-binding proteins such as autophagy adaptors P62 detected ubiquitinated structures on membranes, which in turn recruited mGBPs to the PCVs [104,105]. However, in human cells, ubiquitination and the autophagy adaptor P62 can control *T. gondii* without the need to recruit GBPs [106]. GBPs also directly target microbes present in broken PCVs caused by the activity of microbial secretion systems. Galectin-3 was recruited to the damaged PCVs disrupted by the secretory effectors in HeLa cells infected with *Yersinia enterocolitica*. GBP1 was then recruited to bacteria and interacted with LPS [107]. The roles of GBPs in recognizing and clearing pathogens from vesicles are not fully understood, and further research is needed to differentiate this function between human and mouse cells.

### 4.4. Host Defense Responses Downstream of GBPs Activation

After detecting pathogens, GBPs initiate many cellular defense mechanisms. Assembly of the defense complex in epithelial cells is important for noncanonical inflammasome activation and pyroptosis (Figure 3). Activated caspase-4 cleaves full-length GSDMD to N-terminal GSDMD (N-GSDMD), which is the active form, forming pores on the plasma membrane, leading to pyroptosis [108,109,110]. Moreover, N-GSDMD may directly kill bacteria by disrupting the intracellular and extracellular bacterial OM in vitro. A study found that the depletion of N-GSDMD inhibits the killing of extracellular *Escherichia coli* by pyrolyzed supernatant. In addition, in immortalized mouse bone marrow-derived macrophages and HeLa cells, the killing of *L. monocytogenes* by N-GSDMD has also been observed [111]. Further experiments are needed to determine whether the direct bacterial killing effect of GSDMD is important for controlling infection in vivo. In addition, GBPs take part in the activation of the canonical inflammasomes upon pathogen infection in human monocytes/macrophages. For example, during *Chlamydia* infection, hGBP1 hydrolyzed GTP to GMP, which was further metabolized into uric acid. The resulting uric acid activated NLRP3 inflammasomes [112]. Additionally, studies showed that in *F. novicida*-infected mouse macrophages, mGBP2 and mGBP5 synergistically mediated intracellular killing of bacteria and DNA release, which in turn activated the AIM2 inflammasome [113]. Ultimately, the activated canonical inflammasomes result in caspase-1-dependent pyroptosis accompanied by the secretion of IL-18 and IL-1β, thereby destroying the intracellular replicative niche for bacteria and signaling neighboring cells. Moreover, studies showed that hGBP1 facilitated the disruption of PCVs and *T. gondii* plasma membrane, leading to DNA release, which activated the AIM2 inflammasome and caspase-8-dependent apoptotic cell death [114].

In addition to inducing inflammasome activation and pyroptosis, GBPs can also mediate the destruction of pathogens directly (Figure 3). For example, the binding of GBP1 to *S. flexneri* promoted the circumferential localization of outer membrane protein lcsA, which blocked actin tail formation [101,115]. This inhibits bacterial actin-mediated motility in colonic epithelial cells and cell-to-cell spread. Aside from these, disruption of LPS by GBP1 increased bacterial OM permeability, enabling small bactericidal substances such as apolipoprotein L3 (APOL3) to rupture the inner membranes of pathogenic *Stm* and kill the bacteria directly [102,116]. These mechanisms underscore the multifaceted role of GBPs in host defense.

In summary, GBPs are essential for resisting pathogen infections in both epithelial cells and macrophages. However, their functional outcomes exhibit some differences across these cell types. In epithelial cells, stimulated by LPS, GBPs directly kill invading pathogens or induce noncanonical inflammasome activation, rapidly responding to intracellular pathogens during infection. However, GBPs can display additional functions in macrophages, including activating canonical inflammasome pathways and inducing apoptosis to restrict pathogen proliferation. One possible reason for this is that some proteins highly expressed in macrophages, such as AIM2 and NLRP3, can work together with GBPs during pathogen infections. Despite numerous studies on the structure and function of GBPs in recent years, the specific antimicrobial mechanisms across different pathogens and host cell types have yet to be further explored.

## 5. Recognition of Other Pathogen Components

Beyond nucleic acids and LPS, individual cells can recognize other pathogen components, such as peptidoglycan and flagellin (Figure 1D). For example, on the surface of epithelial cells, PRRs such as TLR2 and TLR5 serve as frontline sentinels. TLR2 recognizes intact peptidoglycan from the bacterial cell wall, while TLR5 detects extracellular flagellin, the structural protein of bacterial flagella [117]. These interactions initiate signaling cascades, which provide molecular preparations for subsequent cell-autonomous defense when pathogens have not yet entered the cell.

Once bacteria or their components enter the cell, some intracellular PRRs can detect their presence. For instance, peptidoglycan-derived fragments, including iE-DAP dipeptide and muramyl dipeptide, are recognized by NOD1 and NOD2 in the cytoplasm, respectively. Activation of these receptors initiates host defense, notably the NF-κB and MAPK pathways, thereby clearing bacterial infections [118,119]. In humans, neuronal apoptosis inhibitory protein (NAIP) can recognize intracellular flagellin and components of the bacterial type III secretion system (T3SS), subsequently activating NLRC4 inflammasomes. In a new study, *Stm* NAIP ligand mutants were delivered to the cytoplasm of a single intestinal epithelial cell in mice using FluidFM technology. The results showed that *Stm* mutants lacking flagellin and T3SS had a reduced capacity to activate the NAIP-NLRC4 inflammasomes [120]. Notably, the application of FluidFM in this research presents an opportunity to explore single-cell defense mechanisms induced by intracellular pathogens.

## 6. Cell-Autonomous Immunity: Implications for Diseases

As stated above, cell-autonomous immunity provides an effective defense against intracellular pathogens. The functional loss or absence of sensors contributes to increased susceptibility to infectious diseases and is often associated with more severe infections. However, activating cell-autonomous defense pathways may also influence the development and occurrence of various non-infectious diseases, which are associated with specific gene mutations or other host-related factors (Figure 2 and Table 2).

### 6.1. Autoinflammatory and Autoimmune Diseases

Mutations in genes encoding proteins involved in cGAS-STING or RIG-I/MDA5-MAVS pathways tend to be strongly linked to autoinflammatory and autoimmune diseases. In 2014, Liu et al. sequenced the *TMEM173* gene, which encodes STING, in patients with symptoms of pulmonary inflammation, cutaneous vasculopathy, and early-onset systemic inflammation. They found that gain-of-function mutations in *TMEM173* were associated with these symptoms. The clinical syndrome is an autoinflammatory disease termed STING-associated vasculopathy with onset in infancy (SAVI). Mechanistically, mutant STING results in constitutive activation of the STING-IFNβ pathway, which leads to excessive IFN production and chronic inflammation. This cell-autonomous activation is a central factor in the pathology of SAVI [122]. Subsequently, additional studies showed that the gain-of-function STING mutation, localized in the Golgi of patient fibroblasts, was also linked to a familial inflammatory syndrome characterized by lupus-like manifestations [123]. A separate study found that mice with a G821S mutation of MDA5 exhibited lupus-like nephritis [153]. It can be seen from the above examples that in the absence of nucleic acid stimulation, mutations of sensors and adaptors still activate downstream signaling, leading to the development of inflammation, which may be related to conformational changes of the mutant proteins.

GBPs have also been implicated in autoinflammatory and autoimmune diseases. The noncanonical inflammasome contributes to sepsis when the initial immune defense is ineffective enough to clear pathogens and a persistent inflammatory response develops [136]. In sepsis caused by severe Gram-negative bacterial infections, blocking the initial hyperinflammatory response is an effective existing therapeutic strategy [154]. Inhibition of GBP-mediated activation of the noncanonical inflammasome may offer a potential approach to reduce mortality. A study has revealed the protective role of GBP1 in inflammatory bowel disease (IBD) [135]. In patients with IBD, GBP1 expression was upregulated at the tight junctions of intestinal epithelial cells. Downregulation of GBP1 via siRNA led to increased apoptosis and decreased barrier function. In addition, GBP5 may play a role in regulating inflammation in some diseases. Research by Haque et al. demonstrated that knockdown of GBP5 in rheumatoid arthritis (RA) synovial fibroblasts exacerbated inflammation and tissue destruction [125]. Consistent with these observations, in a recent study, upregulation of GBP5 was observed in inflamed dental pulp, particularly in human dental pulp stem cells (HDPSCs). The knockdown of GBP5 in HDPSCs significantly increased the expression of some inflammatory cytokines [155].

### 6.2. Cancers

The cGAS-STING signaling pathway also plays a dual role in the development of cancer. Tumor cells release DNA into the cytoplasm due to chromosomal instability, DNA damage, or mtDNA leakage. The released self-DNA initiates the cGAS-STING signaling pathway, which may suppress tumor growth by exerting antiproliferative effects and inducing cell-autonomous death [156,157]. For example, two studies have suggested that the activation of the STING pathway induces apoptosis in malignant B and T cells [158,159]. Moreover, in human osteosarcoma cells, STING formed a transmembrane channel that mediated noncanonical autophagy and cell death [160]. However, other studies showed that tumor-induced aberrant activation of the DNA-sensing pathway was associated with tumor metastasis or survival [161]. For example, the activated cGAS-STING pathway promoted esophageal squamous cell carcinoma (ESCC) tumor growth by inducing autophagy [150]. Similarly, a survival study about triple-negative breast cancer cells also demonstrated the pro-tumorigenic properties of the cGAS-STING pathway [151]. Critical knowledge gaps remain as to why cGAS-STING signaling produces different outcomes on cancer progression. Specifically, it is unclear which key factors tip the balance from an antitumor cell-autonomous immune response toward a pro-tumorigenic state. Addressing these gaps is essential for understanding how the cGAS-STING pathway can be modulated for tumor suppression. Similarly, research on GBPs in cancer reveals conflicting results. Highly expressed GBP1 is associated with decreased progression and good prognosis in certain tumors, for example, breast and colorectal cancer; paradoxically, the opposite is true in others, such as head and neck squamous cell carcinoma and ovarian cancer [162,163,164,165]. The findings emphasize the complexity of the role of GBPs in cancers, and the basis for this disparity is currently unknown.

### 6.3. Clinical Translation and Challenges

Recently, research on inhibitors and agonists targeting nucleic acid-sensing pathways has progressed considerably, offering potential treatments for diseases caused either by insufficient or excessive activation of these pathways. Cyclic dinucleotide (CDN) analogs are the most extensively studied class of STING agonists. Recent clinical studies have shown that CDN analogs such as ADU-S100, MK-1454, and TAK-676 have progressed into phase I and II clinical trials, primarily used for the treatment of various solid tumors [166,167,168]. However, issues related to pharmacokinetics, delivery methods, and species-specific differences have so far constrained their clinical efficacy [169]. STING inhibitors such as indole ureas (H-151) and nitrofurans (C-176 and C-178) targeting the Cys91 residues of STING can inhibit its palmitoylation, thereby preventing overactivation of STING and suppressing the type I IFN response [170,171]. In contrast to STING agonists, cGAS-STING inhibitors remain confined to preclinical research, as issues regarding systemic toxicity and off-target effects call for further refinement to ensure safety and specificity [172,173]. Additionally, dual-specificity phosphatase 4 (DUSP4, also known as MKP2) has recently been shown to inhibit the TBK1-IRF3 pathway downstream of both RIG-I and STING [174]. This finding raises the possibility that targeting DUSP4 could offer a novel means to modulate type I IFN production in relevant diseases. However, most current studies on GBPs have been conducted in vitro and in mouse models in vivo. Therefore, substantial work is required before these findings can be successfully translated into effective treatments for humans.

The burgeoning understanding of cell-autonomous immunity has unveiled promising therapeutic targets for infections, autoimmune diseases, and cancers. However, translating these discoveries of cell-autonomous immunity into clinical therapies faces multifaceted challenges. First, the predictive value of preclinical data is limited due to the discrepancies between animal models and humans. In addition, for infectious diseases, a key challenge lies in balancing the beneficial antiviral or antibacterial responses, which are mediated by pathways such as cGAS-STING and RIG-I/MDA5-MAVS pathways, with the harmful effects of excessive inflammation. In anti-infective therapy, it is critical to clear pathogens effectively while inhibiting an overactive inflammatory response, ensuring the safety and efficacy of the treatment. Moreover, some pathogens have evolved specific effectors to counteract cell-autonomous defenses. For instance, Shigella synthesizes OspC3 and IpaH9.8, which inhibit GBPs-mediated pyroptosis, making it difficult for the host cell to eliminate the pathogen [103]. A deeper investigation into these evasion strategies could facilitate the development of novel therapeutic agents or drug combinations for related infections. Finally, chronic activation of cell-autonomous defense pathways has been implicated in the pathogenesis of many diseases, suggesting that the long-term safety of treatment remains a significant issue. Given the widespread expression of cGAS, RLRs, and GBPs across numerous tissues, systemic administration of their agonists could potentially lead to autoimmune diseases or cytokine storms [175]. Thus, further research is needed to develop precise drug delivery systems that can selectively target infected or tumor cells. Currently, novel drug delivery strategies such as nanocarrier-based delivery and molecular targeting strategies hold promise for selective treatment and reduction of inflammatory side effects [173,176].

## 7. Conclusions and Perspectives

Over the past decade, cell-autonomous defense mechanisms against several kinds of pathogens have become increasingly well understood. The traditional view in immunology posits that the elimination of pathogens is fundamentally dependent on both professional immune cells and cytokine-driven cellular crosstalk. In the scenario of cell-autonomous immunity, non-immune cells are able to clear intracellular pathogens directly without releasing a large number of inflammatory factors to launch complex cell-cell signaling.

However, several questions remain unresolved in this field. The first issue concerns the relationship between metabolism and cell-autonomous immunity. As is known, glucose metabolism, such as glycolysis, is closely related to innate immune processes [177,178,179,180]. Similarly, upon pathogen infection, do epithelial cells exhibit metabolic reprogramming? If so, is this process driven by host-mediated adaptive responses or pathogen-directed subversion? Moreover, to what extent does metabolic regulation contribute to the cell-autonomous defense mechanisms? Another attractive and yet unresolved question is whether non-immune cells exhibit identical responses during primary and secondary pathogen infections. Following the initial pathogen challenge, macrophages and other innate immune cells establish enhanced antimicrobial activity through metabolic adaptation and epigenetic modifications [180,181]. However, whether non-immune cells similarly acquire sustained defensive capabilities that are potentially mediated by epigenetic regulation remains to be fully elucidated. This raises the possibility of “trained immunity” in non-immune cells, wherein prior infection could persistently augment cell-autonomous defense mechanisms.

Currently, autonomous defense mechanisms of non-immune cells remain to be further explored. Emerging tools such as cryo-electron tomography and single-cell manipulation technology allow us to study bacterium-host cell interactions in individual cells more conveniently, which is expected to solve long-standing problems [120,182]. In conclusion, cell-autonomous immunity is an indispensable part of the host defense system against invading pathogens. As this burgeoning field continues to evolve, it provides new perspectives for the treatment of infections and other challenging diseases.

## Figures and Tables

**Table 1:** Nucleic acid sensors in cell-autonomous immunity.

Sensor	Cell Types	Ligand	Signaling Pathways	Downstream Responses	References
cGAS	Macrophage, dendritic cell, fibroblast, epithelial cell	dsDNA	STING-TBK1-IRF3 and IKK-NF-κB pathways	Type I IFN, inflammatory cytokine synthesis	[18,19,20]
DDX41	Macrophage, dendritic cell	dsDNA	STING-TBK1-IRF3 and IKK-NF-κB pathways	Type I IFN, inflammatory cytokines synthesis	[21,22]
IFI16	Macrophage, fibroblast, endothelial cell, epithelial cell	dsDNA	STING-TBK1-IRF3 and IKK-NF-κB pathways, inflammasome activation	Type I IFN, inflammatory cytokines synthesis, pyroptosis	[23,24,25]
AIM2	Macrophage, monocyte, dendritic cell, fibroblast, epithelial cell	dsDNA	Inflammasome activation	Pyroptosis, cytokine secretion	[16,26,27,28]
RNA Pol III	Macrophage, dendritic cell	B-form dsDNA	MAVS-TBK1-IRF3 and IKK-NF-κB pathways	Type I IFN, inflammatory cytokines synthesis	[29,30]
DAI/ZBP1	Macrophage, dendritic cell, fibroblast, epithelial cell	Z-DNA or Z-RNA	TBK1-IRF3 and IKK-NF-κB pathways, RIPK3-MLKL activation, NLRP3 inflammasome activation	Type I IFN, inflammatory cytokine synthesis, PANoptosis	[13,14,31]
RIG-I	Macrophage, dendritic cell, fibroblast, epithelial cell	5′-triphosphate (ppp) short dsRNA	MAVS-TBK1-IRF3 and IKK-NF-κB pathways	Type I IFN, inflammatory cytokines synthesis	[32,33,34,35]
MDA5	Macrophage, dendritic cell, fibroblast, epithelial cell	long dsRNA	MAVS-TBK1-IRF3 and IKK-NF-κB pathways	Type I IFN, inflammatory cytokines synthesis	[36,37,38]
OAS1	Macrophage, monocyte, epithelial cell, microglia	dsRNA	RNase L activation	RNA degradation	[39,40,41,42]
PKR	Macrophage, monocyte, fibroblast, epithelial cell	dsRNA	eIF2α phosphorylation, NF-κB activation	Protein synthesis inhibition, apoptosis, inflammatory cytokine synthesis	[43,44]
NOD2	Macrophage, fibroblast, epithelial cell	ssRNA	MAVS-TBK1-IRF3	Type I IFN, inflammatory cytokines synthesis	[45]
NLRP1	Epithelial cell, keratinocyte	dsRNA	Inflammasome activation	Pyroptosis, cytokine secretion	[46,47]
NLRP6	Epithelial cell, hepatocyte	dsRNA	Inflammasome activation	Pyroptosis, cytokine secretion	[48]
GSDMB	Airway epithelium	dsRNA	MAVS-TBK1 pathway	IFN and ISG expression	[49]

**Table 2:** Cell-autonomous immunity-associated diseases.

Type of Disease	Specific Disease	Mechanism of Disease	References
Autoimmune diseases	Aicardi-Goutières syndrome (AGS)	Mutations in *ADAR1* and three prime repair exonuclease 1 (*TREX1*) lead to hyperactivation of nucleic acid sensors.	[87,121]
	STING-associated vasculopathy with onset in infancy (SAVI)	Gain-of-function mutations in the *TMEM173* gene encoding STING	[122,123]
	Systemic lupus erythematosus (SLE)	Increased cGAS and cGAMP expression	[124]
	Rheumatoid arthritis (RA)	Knockdown of GBP5 in RA synovial fibroblasts exacerbates inflammation and tissue destruction.	[125]
		Elevated levels of cytosolic dsDNA promote inflammatory responses through activation of the cGAS-STING pathway.	[126]
	Singleton-Merten syndrome (SMS)	A gain-of-function *IFIH1* mutation causes SMS.	[127]
	Experimental autoimmune encephalomyelitis (EAE)	The loss of the *MAVS* gene exacerbates the severity of EVE.	[128]
	Multiple sclerosis (MS)	RIG-I and IFIH1 are expressed strongly in patients.	[129]
	Type 1 diabetes	Cumulative effect of *IFIH1* variants and increased *IFIH1* gene expression	[130,131]
	Psoriasis	STING deficiency reduces psoriatic symptoms and inflammation in mouse models of psoriasis.	[132]
	Familial chilblain lupus	Gain-of-function mutations in *STING*	[133]
Inflammatory diseases	Inflammatory bowel disease (IBD)	Loss-of-function mutations in *IFIH1*	[134]
		GBP1 may protect against inflammatory cytokine-induced epithelial apoptosis and the consequent loss of barrier function.	[135]
	Sepsis	Noncanonical inflammasome contributes to sepsis when it fails to clear the infection and a sustained inflammatory response develops.	[136]
	Alcoholic liver disease (ALD)	Ethanol induces ER stress and triggers the interaction between IRF3 and STING.	[137]
	Non-alcoholic fatty liver disease (NAFLD)	cGAS-STING signaling is involved in the development of NAFLD by DNA-mediated type I IFN production.	[138]
		Levels of STING are increased in liver tissues from patients with NAFLD.	[139]
	Acute kidney injury (AKI)	Abnormal cytosolic mtDNA can be sensed by cGAS, resulting in STING-dependent inflammation and renal injury.	[140]
Infectious diseases	*Mycobacterium* infection	*GBP1*^−/−^ mice have difficulty killing *Mycobacterium bovis* BCG through cell-autonomous effects, resulting in an increased bacterial burden.	[141]
		cGAS-deficient mice show increased susceptibility to *Mtb* infection.	[142]
	*L. monocytogenes* infection	The knockdown of IFI16, cGAS, or STING shows reduced induction of IFN expression.	[54]
		*GBP1*^−/−^ mice are susceptible to orogastric infection.	[141]
	*F. novicida* infection	*F. novicida* infection could trigger the cGAS-STING-NLRP3 inflammasome axis in *CASP4* × *TRIF*-deficient human monocytes.	[67]
		GBP2-deficient mice are unable to control *F. novicida* infection.	[113]
	SARS-CoV-2 infection	GBP2 and GBP5 inhibit cleavage of the SARS-CoV-2 spike and reduce viral infections.	[143]
		SARS-CoV-2 infection triggers cGAS-STING signaling in endothelial cells via the release of mtDNA, resulting in cell death and type I IFN production.	[59]
	HSV infection	cGAS-deficient mice are more susceptible to lethal infection with HSV-1.	[50]
	CMV infection	Type I IFN expression is essentially abolished in STING-deficient endothelial cells after CMV infection.	[64]
	Influenza virus infection	Loss-of-function mutations in *DDX58*, which encodes the RIG-I receptor, correlate with high susceptibility to respiratory infections caused by the influenza virus.	[144]
	*Plasmodium* infection	Genomic DNA from *Plasmodium falciparum* may access the cytosol due to phagosomal destabilization and activate type I IFN in Malaria.	[145]
	*T. gondii* infection	Gbp^chr3^-deficient mice exhibit increased susceptibility to *T. gondii* infection.	[146]
Cancers	Colorectal cancer	The absence of STING leads to excessive colon inflammation during the early stages of tumor development, and STING-deficient mice are highly susceptible to colorectal cancer.	[147]
	Glioma	Hypermethylation of the STING promoter mediates STING silencing in glioblastoma, contributing to immune suppression.	[148]
		GBP1 promotes EGFR-mediated MMP1 expression, thereby enhancing glioma cell invasion.	[149]
	Esophageal squamous cell carcinoma (ESCC)	The mtDNA stress activates the cGAS-STING pathway, promoting autophagy and ESCC progression.	[150]
	Breast cancer	The cGAS-STING drives the IL-6-dependent survival of triple-negative breast cancer.	[151]
		GBP2 inhibits Drp1-mediated mitochondrial fission to suppress breast cancer invasion.	[152]
